# Supplementary figures and images for: Big data: Airway management at a university hospital over 16 years; a retrospective analysis
Source: PLoS One. 2022 Sep 20;17(9):e0273549. doi: 10.1371/journal.pone.0273549 (PMC9488754; doi:10.1371/journal.pone.0273549)

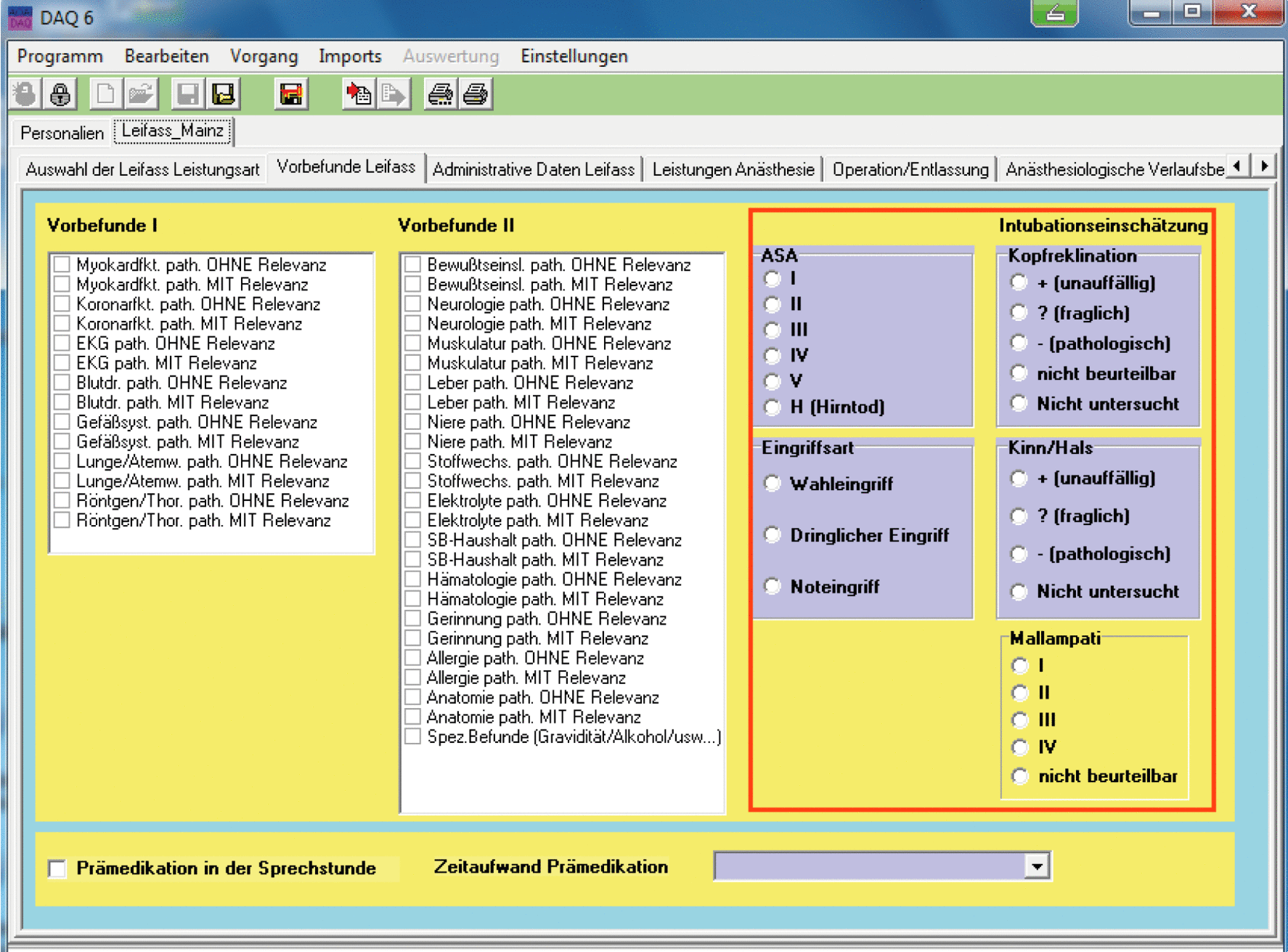

Supplement: S1 Fig — Purple boxes are mandatory fields. English translation of relevant text elements (red box): Hirntod = brain death, Eingriffsart = type of surgery, Wahleingriff = elective, dringlicher Eingriff = urgent, Noteingriff = emergency, Kopfreklination = neck reclination, unauffällig = normal, fraglich = questionable, pathologisch = pathological, nicht beurteilbar = not evaluated, nicht untersucht = not analysed, Kinn/Hals = thyromental distance. (GIF) [file pone.0273549.s001.gif]

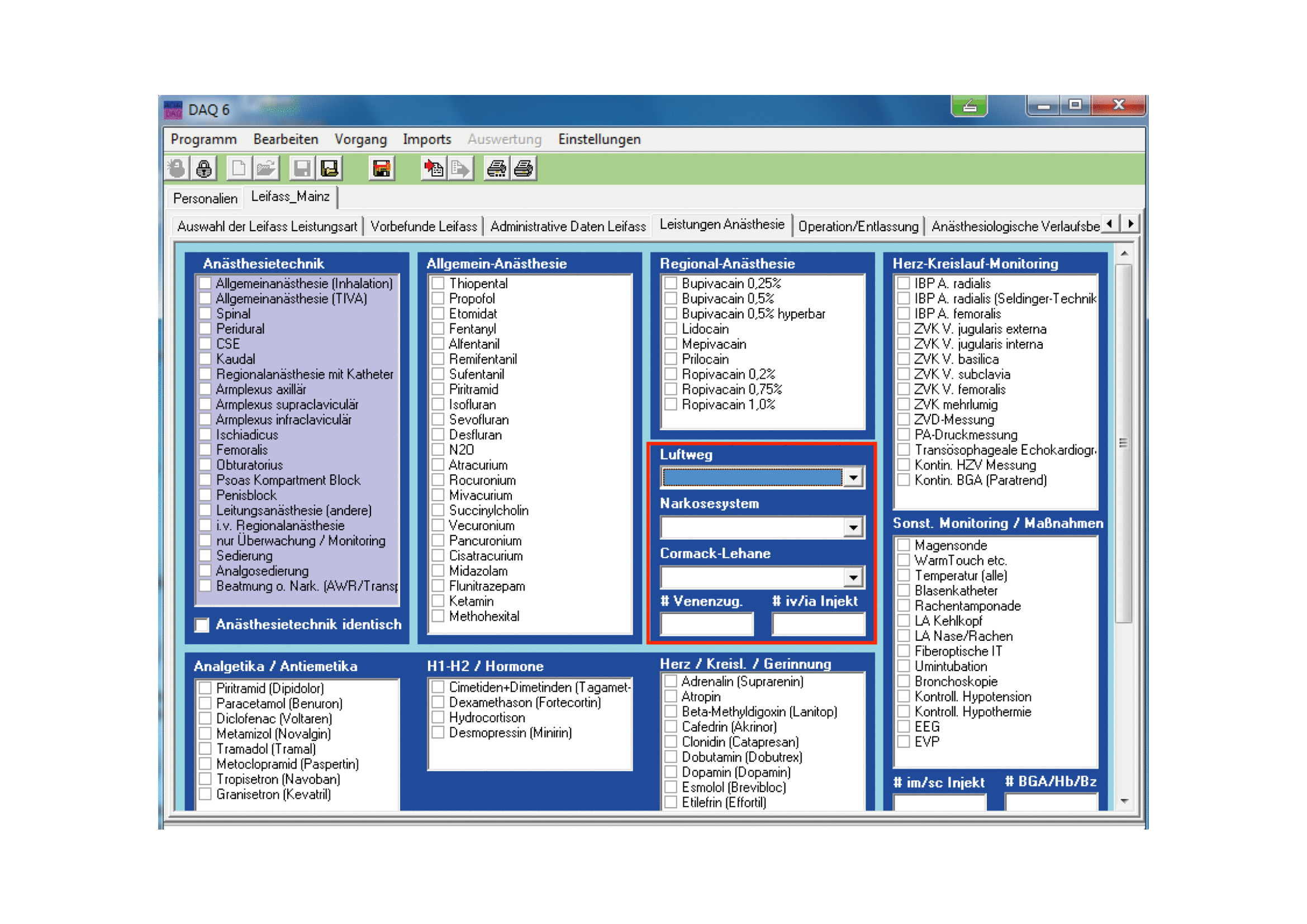

Supplement: S2 Fig — English translation of relevant text elements (red box): Luftweg = airway management technique. (GIF) [file pone.0273549.s002.gif]
