# Supplementary material for: Big data: Airway management at a university hospital over 16 years; a retrospective analysis
Source: PLoS One. 2022 Sep 20;17(9):e0273549. doi: 10.1371/journal.pone.0273549 (PMC9488754; doi:10.1371/journal.pone.0273549)
Supplement: Attachment — Submitted filename: Peer review PONE.docx [file pone.0273549.s007.docx]

Peer review PONE

Big data: Airway management at a university hospital over 16 years; a retrospective analysis

In the age of ever-increasing knowledge regarding airway management, to know details and trends in time about the procedures, techniques and uses of the approaches remains very useful. I appreciate the opportunity to carefully read and comment regarding the submission “Big data: Airway management at a university hospital over 16 years; a retrospective analysis”. The idea and the availability of data to examining the trends over time in such a big number of procedures is commendable and with major clinical application.

Major comments,

- Most analytic goals for studies can be viewed as either seeking evidence of causality or prediction. From my early view, I feel you really present a descriptive analysis, and this is wonderful. I really congratulate about that. Many people think if they are not doing exploratory analysis o some related are not doing good science and that is a big mistake (<https://doi.org/10.1038/s41416-020-1019-z>). Research studies that focus on describing a population of interest are essential building blocks for both causal and predictive frameworks, and do not typically require control for additional variables. To understand the purpose of a study (i.e., descriptive, causal, or predictive), it is vital that the goals of the research be clearly explained. Please consider adjusting your purposes section of the abstract to become it more transparent and clearer (see below).
- Please, do not emphasize in the whole text any analysis on the basis of confounding. Here, I can see a great use of descriptive epidemiology beyond any causal purpose.

Minor comments per section,

Abstract

- I find the abstract quite difficult to understand. First, the purpose does not clarify in detail the aims of this study limiting itself to the need of data in this area. Please, consider clarifying the aims of this study at the end of the first section: “purpose”.
- Let’s consider adding to the methods section if you are including only adults, children, pregnant patients, etc.
- In the results section, please avoid the use of only significance testing during reporting of results. Quote “Over time, second generation laryngeal masks were used more frequently than first-generation laryngeal masks (p < 0.001)” can be adjusted by adding the degree of reduction over time, or at least over two extreme years.
- The conclusion seems to be a repetition of the results. Please conclude by following your results, perhaps regarding the trends directly and the reduction on the use of certain techniques over time.

Introduction

- It is clear the aims of your study after reading the full introduction.

Methods

- It is quite impressive that in such amount of data you do not have missing values. Methods section describes very well the process of data collection from the system, but it does not mention at all any relevant information for potential information bias from that system, how did you address that? is there any potential variable to be missed or at least biased?
- Following the previous comment, STROBE request information about how missing data or potential biases were addressed. It is quite relevant to provide information about to enhance completeness of you report. Please add relevant information.
- Results section mention “For 15,756 anaesthesia patients (4.6%) the type of procedure was not evaluated.”. Please, would you clarify why? Are those patients considered as missing values from the analysis?
- I may understand you used all data from your collection system. Anyway, it is relevant ot provide information to reader about sample size. It was not calculated so that should be stated and mentioned. Explain how the study size was arrived at?

Results

- Study cohort and characteristics. Always it is a good idea to provide a Flowchart regarding data sources and inclusion/exclusion criteria used. Indeed, completeness checklist includes a flowchart diagram as initial part of results section. Please, consider adding that flowchart of patient selection and inclusion. Report numbers of individuals at each stage of study—eg. numbers potentially eligible, examined for eligibility, confirmed eligible, included in the study, completing follow-up, and analyzed
- Figures 1-6 are very well presented and attractive. Please add all relevant information to each Figure to the reader, including colors.

Discussion

- While I agree with this point “To our knowledge, there are only few studies with a comparable large data set of …”, I would like to add also this fact reduces external validity of your results. I do not see any section of the discussion taking about the type of patients in terms of complexity you receive in your center. Are those patients reflecting the common clinical practice in other health care systems? In what extent? Please, expand about that and discuss the generalizability (external validity) of the study results
- Discussion is very well conducted and interesting enough. To me, as a reader it was very nice to explore.
- You mention clear limitations. However, measurement error was not included explicitly. Quite complex in retrospective datasets. Any potential to misclassify exposure, outcome or included covariates. Any consideration about steps taken to ensure accurate measurement (double extraction, double check). Thank you for expanding on these details.
- You mention several times in the text the importance of this data to clinical practice guidelines. Quote “…and serve as a basis for future adaptation of airway management guidelines.”. Do your consider people in your hospital or perhaps country is following current guidelines in airway management and that is reflected in your data? I would like to suggest citing and mention that current guidelines in airway management seems to be well developed but poorly able to be adapted to clinical practice (CPGs show low applicability of their recommendations to real clinical practice) (<https://doi.org/10.1097/eja.0000000000001195> ). Please, could you expand on this topic perhaps a final paragraph.
