# Supplementary material for: Big data: Airway management at a university hospital over 16 years; a retrospective analysis
Source: PLoS One. 2022 Sep 20;17(9):e0273549. doi: 10.1371/journal.pone.0273549 (PMC9488754; doi:10.1371/journal.pone.0273549)
Supplement: Attachment — Submitted filename: Response to Reviewers.docx [file pone.0273549.s008.docx]

| To the Editors and Reviewers of  PLOS ONE | Dr. med. Nina Pirlich  Consultant Anaesthetist  Head of the research group „Airway management“  Langenbeckstr. 1  55131 Mainz  Telefon: +49 (0) 6131 17- 2854  E-Mail: pirlich@uni-mainz.de |
| --- | --- |

Mainz, 19 July 2022

*Rebuttal Letter*

*Big data: Airway management at a university hospital over 16 years; a retrospective analysis*

Dear Editors,

Dear Reviewers,

we thank you very much for your efforts in having our paper reviewed and for giving us the opportunity to revise it. We did our best to comprehensively address all the reviewers’ comments.

Please find attached the list of changes of each point which has been commented.

We hope to have revised this paper to your satisfaction and are looking forward to hearing back from you.

Sincerely,

Dr. med. Nina Pirlich

Department of Anaesthesiology

University Medical Center Mainz

**List of changes concerning Reviewers' comments:**

**Reviewer #1:** Peer review PONE
Major comments,
• Most analytic goals for studies can be viewed as either seeking evidence of causality or prediction. From my early view, I feel you really present a descriptive analysis, and this is wonderful. I really congratulate about that. Many people think if they are not doing exploratory analysis or some related are not doing good science and that is a big mistake (<https://doi.org/10.1038/s41416-020-1019-z>). Research studies that focus on describing a population of interest are essential building blocks for both causal and predictive frameworks, and do not typically require control for additional variables. To understand the purpose of a study (i.e., descriptive, causal, or predictive), it is vital that the goals of the research be clearly explained. Please consider adjusting your purposes section of the abstract to become it more transparent and clearer (see below).

We thank the reviewer for the positive assessment of our analysis strategy.

• Please, do not emphasize in the whole text any analysis on the basis of confounding. Here, I can see a great use of descriptive epidemiology beyond any causal purpose.
As explained below, we have adjusted the wording to convey the aims of our study more precisely.

Minor comments per section,
Abstract
• I find the abstract quite difficult to understand. First, the purpose does not clarify in detail the aims of this study limiting itself to the need of data in this area. Please, consider clarifying the aims of this study at the end of the first section: “purpose”.

We have tried to clarify the aims of this study at the end of the first section: "purpose" (p.3, line 24 29)

• Let’s consider adding to the methods section if you are including only adults, children, pregnant patients, etc.

We highlighted more clearly in the methods section that no exclusion criteria were applied with respect to patient characteristics. We analyzed every patient who underwent a surgical or medical procedure with anaesthesiological care at our tertiary care facility (p. 3, line 33 -34).
• In the results section, please avoid the use of only significance testing during reporting of results. Quote “Over time, second generation laryngeal masks were used more frequently than first-generation laryngeal masks (p < 0.001)” can be adjusted by adding the degree of reduction over time, or at least over two extreme years.

We have eliminated the use of p-values to indicate statistical significance. We now focus an estimating group differences and time trends including their uncertainty.

• The conclusion seems to be a repetition of the results. Please conclude by following your results, perhaps regarding the trends directly and the reduction on the use of certain techniques over time.
The conclusion now was drawn following the results with a direct focus on the trends. (p. 4, line 55 – 57)

Introduction
• It is clear the aims of your study after reading the full introduction.

Methods
• It is quite impressive that in such amount of data you do not have missing values. Methods section describes very well the process of data collection from the system, but it does not mention at all any relevant information for potential information bias from that system, how did you address that? is there any potential variable to be missed or at least biased?

The input into the DAQ was performed by all employees who have worked at the Department of Anaesthesiology over the 16 years. Individual bias can therefore be excluded. The data set can only be finalized for each patient if all the required information is entered completely, otherwise no billing can take place. This ensures that all datasets are completed, and no relevant information is missing.

We only evaluated those variables that were available in the DAQ. In retrospect, more detailed information on the use of videolaryngoscopy would have been of interest. This problem was pointed out in the discussion.

• Following the previous comment, STROBE request information about how missing data or potential biases were addressed. It is quite relevant to provide information about to enhance completeness of you report. Please add relevant information.

Please find the added information in the previous comment.
• Results section mention “For 15,756 anaesthesia patients (4.6%) the type of procedure was not evaluated.”. Please, would you clarify why? Are those patients considered as missing values from the analysis?

The DAQ system allows the input "not evaluated" when selecting the type of airway procedure. Those patients are included in the analysis as „not evaluated“.
• I may understand you used all data from your collection system. Anyway, it is relevant to provide information to reader about sample size. It was not calculated so that should be stated and mentioned. Explain how the study size was arrived at?

No formal sample size requirements were calculated before conducting the study. It was the intention to analyze the complete available data from the internal data base from our center to report on long term trends We added this information in the material and method section. (p. 7, line 107-109)

Results
• Study cohort and characteristics. Always it is a good idea to provide a Flowchart regarding data sources and inclusion/exclusion criteria used. Indeed, completeness checklist includes a flowchart diagram as initial part of results section. Please, consider adding that flowchart of patient selection and inclusion. Report numbers of individuals at each stage of study—e.g., numbers potentially eligible, examined for eligibility, confirmed eligible, included in the study, completing follow-up, and analyzed

There was no patient selection and exclusion. Data from all patients requiring surgery or medical intervention with anaesthesiologic attendance within a period of 16 years were analysed. There was also no follow-up.

• Figures 1-6 are very well presented and attractive. Please add all relevant information to each Figure to the reader, including colors.
All relevant information is presented in detail in the legends of the figures.
Discussion
• While I agree with this point “To our knowledge, there are only few studies with a comparable large data set of …”, I would like to add also this fact reduces external validity of your results. I do not see any section of the discussion taking about the type of patients in terms of complexity you receive in your center. Are those patients reflecting the common clinical practice in other health care systems? In what extent? Please, expand about that and discuss the generalizability (external validity) of the study results

Due to the presence of a large department of oral and maxillofacial surgery and a large otorhinolaryngology, we treat patients with an exceptional number of difficult airways. Patients with large tumors and abscesses in the airway and patients with rare syndromal and anatomical peculiarities belong to our complex patient population. Due to this condition, the results of this analysis cannot be transferred to other health care institutions. (Added on p 24, line 345 – 349)

• Discussion is very well conducted and interesting enough. To me, as a reader it was very nice to explore.
• You mention clear limitations. However, measurement error was not included explicitly. Quite complex in retrospective datasets. Any potential to misclassify exposure, outcome or included covariates. Any consideration about steps taken to ensure accurate measurement (double extraction, double check). Thank you for expanding on these details.

We appreciate the reviewer’s concern for high data quality. We have added an assessment of the data quality. In short, variables concerning patient demographics and frequencies of airway management techniques can be objectively evaluated and are very unlikely to contain misclassification errors. In particular, data extraction procedures from the internal documentation data base were automated to avoid errors from manual repetitive tasks. However, evaluation of patient characteristics such as ASA status, Mallampati score, or Cormack-Lehane score has limited reliability, and thus has potentially higher probability of misclassification. (p. 24, line 3*52*-357)

• You mention several times in the text the importance of this data to clinical practice guidelines. Quote “…and serve as a basis for future adaptation of airway management guidelines.”. Do your consider people in your hospital or perhaps country is following current guidelines in airway management and that is reflected in your data? I would like to suggest citing and mention that current guidelines in airway management seems to be well developed but poorly able to be adapted to clinical practice (CPGs show low applicability of their recommendations to real clinical practice) (<https://doi.org/10.1097/eja.0000000000001195> ). Please, could you expand on this topic perhaps a final paragraph.

In fact, we recently conducted an online survey on the current practice of German airway management with the main outcome that anesthesiologists mostly adhered to the German guidelines. [Pirlich N, Dutz M, Wittenmeier E, Kriege M, Didion N, Ott T, et al. Current practice of German anesthesiologists in airway management: Results of a national online survey. Anaesthesist. 2021.] We have referred to this in the introduction (p.5, line 75 - 77).

In agreement with your proposal, we have added a paragraph on the applicability of guidelines and their recommendations in clinical practice:

„Guidelines are intended to ensure a high degree of patient safety and at the same time recommend a corridor of action for the treating anesthesiologist. It is useful and necessary to adapt the respective guidelines to one's own everyday life and the existing circumstances. The clinical (regional or local) context should be considered as an important factor for enhancing adherence to the recommendations by physicians and health systems [Merchan-Galvis AM, Caicedo JP, Valencia-Payan CJ, Calvache JA. Methodological quality and transparency of clinical practice guidelines for difficult airway management using the appraisal of guidelines research & evaluation II instrument: A systematic review. Eur J Anaesthesiol. 2020;37(6):451-6.]. (p. 25, line 365 – 369)

**Reviewer #2:** This is an interesting well written manuscript that in my opinion is adequate for publication after minor revision.
I only have two comments:
In limitations it should be mentioned that data comes from single hospital, so the external validity is limited.

Adding to the limitations on page 24 (line 350-351), we have now added one more sentence on this subject: „External validity is therefore limited.“

Study period goes until just before the COVID 19 pandemic started. Some comment on the effect of the pandemic on the results could be interesting.

We thank you for this indeed very interesting aspect you mention here.

As soon as the data review of the last years is completed, we will perform an evaluation of the pandemic years to identify possible effects on airway management.

**Reviewer #3:** I would like to commend the authors for looking at 16 years of data and in excess of 340,000 airway management procedures. This is big data.

Specific comments:

Page 5 line 50-64: This portion may not be immediately relevant to the study and can be shortened or summarized.

We have slightly condensed the introduction. However, we argue that carefully explaining the current state of research is necessary to understand the motivation and aims of our study.

Page 7 line 93: Cormack and Lehane grading may not be airway evaluation data but rather intubation grading data.

The term "intubation grading data" was included.

Page 10 line 150: Data from 414,843 patients were analyzed but only 340748 patients were included. Could the authors explain in more detail why this was so?
Data from 414,843 patients include every patient, undergoing an operation or medical intervention with anaesthesiologic attendance. Anaesthesiologic assistance includes procedures without securing the airway, like stand-by, sedation, regional and local anaesthesia. 340,748 patients received an airway procedure.

General comments about the intrinsic limitations of the study as presented by the authors:

1. 16 years ago, airway management may be different. Use of 2nd generation supraglottic airway devices and video laryngoscopes more widespread in the last decade. I wonder if limiting the period to 10 years may be more relevant to practicing Anaesthesiologists.

We agree that airway management has changed a lot over the 16-year period. We have tried to demonstrate this by showing trends over time.By analyzing a longer time frame, we hope to highlight the substantial changes in recent years.

2. Use of laryngeal mask only 27% in 2020. That figure seems a little low and is not in keeping with international data and my own experience. It should be closer to 50%, of course depending on the type of cases that are performed.
One possible reason for the low use of laryngeal masks in our analysis could be the more complex surgical procedures in a university setting that require tracheal intubation. Overall, it must be assumed that the national and institutional differences in the use of laryngeal masks seem to be influenced by an emotional discussion about the “safe” airway (intubation) and the “unsafe” airway (laryngeal mask).

3. One main finding is that laryngeal mask use has increased over time. This has been very well reported in the literature. It would be interesting to know if the use of video laryngoscopes has likewise increased over time.

As we state in our limitations, we unfortunately do not have data on the use of videolaryngoscopy.

4. A more interesting slant may also be a subgroup analysis of the use of fibreoptic intubation, e.g., indications, complications, failure rate, etc.
This is exactly what we planned.

5. There are generally too many figures. These should be reduced.
We have decided to abandon Figure 5 and Figure 6.

6. Conclusions by the author are not novel and many (if not all) of these have been espoused in other prior publications or are intuitive.

Minor comment:

1. Consider using the term supraglottic airway device instead of laryngeal mask (which is a brand name). Consider detailing the types of supraglottic airway devices used during the 16-year period (e.g. Ambu AuraGain, LMA Protector, Igel, etc)

The term „laryngeal mask“ has been replaced with „supraglottic airway device“ throughout this document. Detailed types of supraglottic airway devices are now listed on page 9, line 141-143.
